# Precision Intervention of Isorhamnetin Total Flavonoids in Ischemic Heart Failure: Mechanistic Exploration Based on Signature Gene Targets

**DOI:** 10.3390/cimb48040406

**Published:** 2026-04-15

**Authors:** Li-Juan Zhang, Xu Hu, Kader Kaderyea, Wen-Ling Su, Yue Wang, Di-Wei Liu, Rui-Fang Zheng, Jian-Guo Xing

**Affiliations:** 1 School of Pharmacy, Xinjiang Medical University, Urumqi 830017, China; 2229840722@stu.xjmu.edu.cn (L.-J.Z.); kaderya0303@163.com (K.K.); yws990715@163.com (W.-L.S.); wangyue20165643@163.com (Y.W.); 2 Xinjiang Key Laboratory of Uyghur Medical Research, Xinjiang Institute of Materia Medica, Urumqi 830004, China; yaki_hx@163.com (X.H.); liu3498921775@163.com (D.-W.L.); 3 Department of Clinical Pharmacy, School of Preclinical Medicine and Clinical Pharmacy, China Pharmaceutical University, Nanjing 211198, China

**Keywords:** *Dracocephalum moldavica* L., ischemic heart failure, LASSO, Random Forest, molecular docking, total flavonoid

## Abstract

Early identification of ischemic heart failure (IHF) is critical for improving patient prognosis and clinical outcomes. However, effective diagnostic biomarkers and targeted therapeutic strategies for IHF remain limited. Total flavonoids from *Dracocephalum moldavica* L. (TFDM) exert potential cardioprotective effects; however, the molecular mechanisms by which TFDM acts against IHF have not been fully elucidated. Therefore, this study aims to identify diagnostic biomarkers for IHF and explore the potential therapeutic mechanism of TFDM targeting these key genes. Given the small sample size (*n* = 17) of the clinical dataset, LASSO regression and Random Forest were employed due to their superior performance in feature selection, noise reduction, and stability in small-sample scenarios. In this study, we screened key characteristic genes of IHF through bioinformatics analysis and further investigated the binding potential between these key genes and active components of TFDM using molecular docking, thus providing new targets for the early diagnosis of IHF and new evidence for the intervention mechanism of TFDM in IHF.

## 1. Introduction

Heart failure (HF) is a complex clinical condition defined as the heart’s inability to pump blood effectively due to a variety of etiologies that lead to changes in myocardial structure and function, resulting in impaired ventricular filling and ejection. This condition not only poses a serious threat to patients’ lives but also represents a major economic challenge for the affected families and broader society [1,2].

Defined by its specific etiology, IHF is characterized by coronary artery blockage due to atherosclerotic plaques. This leads to prolonged ischemia and hypoxia of the myocardium, subsequently causing myocardial cell death and conditions such as myocardial fibrosis, ultimately resulting in a decline in cardiac function and the onset of heart failure. This condition often manifests in the advanced stages of coronary artery disease and can easily trigger symptoms such as arrhythmias, leading to patient death within a relatively short period [3,4].

Conventional treatment measures for IHF often involve vasodilation, enhancement of the blood oxygen content, and antithrombotic therapies. However, these measures do not improve the underlying condition of increased oxygen demand in the body. Therefore, while patients undergoing conventional treatment may survive if promptly rescued, their hearts may sustain severe damage, which is detrimental to their future quality of life [5,6]. Consequently, early diagnosis and timely effective intervention are crucial for improving the prognosis of patients with IHF and reducing the associated mortality rates [7,8].

*Dracocephalum moldavica* L. is a traditional Uyghur medicinal herb with potential therapeutic effects on cardiovascular diseases. TFDM is the main active component extracted from it, but its specific chemical composition and mechanism of action in the treatment of IHF remain unclear. The qualitative analysis of TFDM is an essential prerequisite for exploring its pharmacological effects. Therefore, this study first employed HPLC-HRMS technology to identify TDFM’s potential active components. Then, multiple bioinformatics approaches were used to identify characteristic genes with significant diagnostic efficacy for IHF, to characterize the functional enrichment profiles of relevant signaling pathways, and to investigate the interactions between their protein targets and TFDM’s active ingredients, aiming to provide new perspectives for clinical diagnosis and treatment.

## 2. Materials and Methods

### 2.1. Qualitative Analysis of TFDM

#### 2.1.1. Instruments and Materials

The following instruments were used: Thermo Ultimate 3000 high-performance liquid chromatograph, LTQ Orbitrap XL high-resolution mass spectrometer (USA), and IQ7000 ultrapure water machine (USA). The TFDM sample was provided by the (China).

#### 2.1.2. Preparation of Sample Solution

To obtain the TFDM sample solution, we took approximately 200 mg of TFDM powder, placed it in a stoppered conical flask, accurately added 20 mL of 80% methanol, shook the resulting solution well, and let it stand for 40 min. Then, the solution was filtered through a 0.45 μm microporous membrane, and the subsequent filtrate was taken as the sample solution.

#### 2.1.3. Experimental Conditions

The chromatographic conditions were as follows: SUPERIOREX ODS C18 chromatographic column (250 mm × 4.6 mm, 5 μm); mobile phase A: 0.1% formic acid–water, mobile phase B: acetonitrile, gradient elution (Table 1); flow rate: 1.0 mL/min; column temperature: 25 °C; detection wavelength: 310 nm; injection volume: 10 μL. The mass spectrometry conditions were as follows: electrospray ionization source (ESI), positive and negative ion mode scanning, high-resolution data acquisition (HRMS), resolution 30,000; ion transfer tube temperature: 375 °C; spray voltage: 4.5 kV; ion transfer tube voltage: 60 V; sleeve lens voltage: 80 V; sheath gas: 30 arb; auxiliary gas: 10 arb; purge gas: 0 arb; full scan mode (Full Scan), scanning range *m*/*z* 50~2000; secondary ions adopt data-dependent scanning, collision-induced dissociation (CID) data acquisition, CID normalized energy 35%.

#### 2.1.4. Data Analysis

The obtained mass spectrometry data were analyzed using Compound Discoverer 3.3 data processing and analysis software, and the relevant natural product databases were used to search for possible substances in the mass spectrometry data. The minimum mass spectrometry response value was set to 10 × 10^6^, and the mass spectrometry molecular weight deviation was less than 5 ppm. The retrieved data were filtered to obtain the analysis results under the positive and negative ion modes of the ESI source.

### 2.2. Data Sources

The dataset GSE26887 (Platform: GPL6244) was retrieved from the Gene Expression Omnibus (GEO) as the training cohort. It comprised 17 subjects, with 12 IHF patients and 5 normal controls.

### 2.3. Identification of DEGs

The limma R package v4.1.2. [9] was employed to identify the DEGs (|logFC| > 1, *p* < 0.05) between IHF patients and controls. The resulting DEGs were graphically represented in a volcano plot, with a heatmap further highlighting the top 50 upregulated and downregulated candidates.

### 2.4. Functional Annotation and Enrichment

The candidate DEGs were subjected to GO and KEGG functional annotation utilizing the clusterProfiler R package v4.1.2. [10]. GO enrichment was used to systematically evaluate the functional attributes of DEGs across the standard domains: biological processes, cellular components, and molecular functions. KEGG pathway enrichment analysis was conducted to elucidate the underlying mechanisms.

### 2.5. Co-Expression Network Construction

In accordance with scale-free topology principles, a WGCNA was conducted using the GSE26887 transcriptome data [11]. The pickSoftThreshold function within the WGCNA package was used to calculate the soft thresholding indices and adjacency matrices. To proceed, the adjacency matrices were converted into topological overlap matrices (TOMs), and corresponding degree measures were calculated. To identify co-expressed modules, dynamic tree cutting (min. module size = 50) was applied to the hierarchical clustering results. The relevance of each gene module to IHF was assessed via quantified gene significance (GS) and module membership (MM), thereby defining pivotal modules for further analysis.

### 2.6. Determination of Gene Signature

An integrated analysis, combining DEGs and co-expression modules, pinpointed core genes through feature selection with LASSO and the Random Forest algorithm.

A dual-machine learning strategy was employed for robust gene selection. LASSO regression with 10-fold cross-validation (glmnet package) handled high dimensionality [12], while a Random Forest model (randomForest package) evaluated feature importance. The final model, tuned to the optimal number of trees (assessed across 1–500 iterations), selected hub genes with an importance score exceeding 0.5 [13].

This study identified the genes jointly selected by the two machine learning algorithms as characteristic genes for patients with IHF. The diagnostic performance of the gene signature was quantified by the area under the ROC curve (AUC), with values above 0.7 denoting good discriminative ability.

### 2.7. Functional Interpretation of Gene Sets

GSEA was applied to reveal the signaling pathways linked to the hub genes by comparing sample subgroups defined by median expression (significance: FDR < 0.05) [14].

### 2.8. Molecular Docking

We conducted molecular docking analysis between TFDM’s active components and four protein targets (CTRP9, CCR1, CD163, P2Y13). The chemical constituents of *Artemisia argyi* were obtained through literature searches, and candidate compounds were sourced from the BATMAN-TCM and ChemSrc databases, followed by pharmacokinetic screening employing the SwissADME platform [15,16]. For these searches, the following criteria were applied: gastrointestinal absorption must be “high”; with a bioavailability score ≥ 0.5; compliance with at least two drug likeness rules; and restriction to flavonoid compounds.

The three-dimensional structural information of the structural coordinates for the proteins of interest was acquired from the RCSB PDB with PDB IDs 7VL9, 6K0O, B2RNN3, and Q9BPV8, respectively. Prior to molecular docking, we utilized the Discovery Studio software https://www.3ds.com/products/biovia/discovery-studio/ (accessed on 1 February 2026) to remove crystallographic solvent molecules and non-covalent ligands and employed the AutoDock Vina software for hydrogen atom addition and electron supplementation of the protein structures.

Compound structures in SDF format were obtained from PubChem and subjected to similar processing using the AutoDock Vina software, including hydrogen atom addition, electron supplementation, and the addition of rotatable bonds and torsions to ensure flexibility and accuracy during the docking process.

For the molecular docking procedure, the protein structures were set as rigid bodies, and the docking algorithm was executed to identify and record the top three conformations with the lowest binding energies for each protein. These conformations allowed us to assess the binding affinity of the compounds to their protein targets. Subsequently, the optimal molecular docking results were visualized using the Pymol software.

To further investigate the interaction details between the active compound molecules and the CTRP9, CCR1, CD163 and P2Y13 target proteins, we performed a detailed analysis of the molecular docking structures using the Discovery Studio software. This included the identification and evaluation of potential hydrogen bonds, hydrophobic interactions, van der Waals forces, and other non-covalent interactions, thereby providing crucial information for understanding the biological activity of the compounds and their potential mechanisms of drug action.

### 2.9. In Vitro Validation of Machine Learning Screening Candidates for Pharmacological Targets

#### 2.9.1. Materials and Methods

##### Animals

This study utilized 40 Male Sprague Dawley (SD) rats aged 2–3 months with a body weight of 260 ± 20 g, purchased from Henan Skebes Biotechnology Co., Ltd. [Production License No.: SCXK(Jing)2016-0010]. All rats were housed in the SPF-grade animal facility of the Xinjiang Institute of Pharmaceutical Research under standard conditions, maintained at controlled temperature and humidity with a 12-h light–dark cycle. Prior to formal experiments, animals underwent one week of acclimatization in a controlled environment at 24–26 °C with free access to food and water. This study protocol was approved by the Animal Ethics Committee of the Xinjiang Institute of Materia Medica (Ethics Approval No.: XJIMM–20240403), and all procedures strictly adhered to animal ethics guidelines.

##### Drugs and Reagents

TFDM (purity: 60.60–62.35%, Batch No.: 20180406) was self-prepared at the Xinjiang Institute of Materia Medica. The rat Creatine Kinase MB Isoenzyme (CK-MB) ELISA Kit (Batch No.: E-EL-R1253) was purchased from Nanjing Jiancheng Bioengineering Institute. The rat Cardiac Troponin I (TNNI3/cTn-I) ELISA Kit (Batch No.: E-EL-R1253) was obtained from Elabscience Biotechnology Co., Ltd. The CTRP9 antibody (Batch No.: A00081-03-100; Brand: Anitbody) was acquired. The EasyScript One-Step gDNA Removal and cDNA Synthesis SuperMix (Batch No.: S10112) was procured from TransGen Biotech Co., Ltd. (Beijing). The P2Y13 antibody (Batch No.: ab108444; Brand: abcam) and the CCR1 antibody (Batch No.: A18341; Brand: ABclonal) were used. GAPDH antibody, HRP-labeled Goat Anti-Rabbit IgG antibody, and HRP-labeled Goat Anti-Mouse IgG antibody were purchased from ZSGB-BIO. A hypersensitive ECL chemiluminescent substrate (BOSTER) and 0.45 μm PVDF membranes (Millipore) were also used.

##### Instruments

The following instruments were used: a multifunction imaging system (FUSION Fx6; VILBER, France); electrophoresis power supply (PowerPac™ Universal Power Supply) and electrophoresis tank (Bio-Rad, USA); an ultrasonic cell disruptor (Model JY92-IIN; Ningbo Scientz Biotechnology Co., Ltd.); a benchtop high-speed refrigerated centrifuge (Model TGL-16k; Hunan Xiangyi Laboratory Instrument Development Co., Ltd.); a multifunction microplate reader (Model SPARK; TECAN SPARK, Switzerland); an electric thermostatic blast drying oven (Model DGG-9240A; Shanghai Qixin Scientific Instrument Co., Ltd.); a real-time fluorescence quantitative PCR system (Model QuantStudio 3; Applied Biosystems, USA); an ice maker (Hangzhou Zhongleng Electrical Co., Ltd.); a vortex mixer (Qilinbeier Instrument Manufacturing Co., Ltd., Haimen, China); and micropipettes (Eppendorf AG, Germany).

#### 2.9.2. Methods

##### Model Establishment and Grouping

Forty SD rats were randomly allocated to Control, MIRI model, Total Flavonoids from *Dracocephalum moldavica* L. treatment (*n* = 10 per group) (MIRI + TFDM, 180 mg·kg^−1^), and Compound Danshen Dripping Pills (MIRI + Y, 75 mg·kg^−1^) groups. After one week of acclimatization, the rats were pre-treated with TFDM via intragastric administration for one consecutive week. The control and model groups served as vehicle controls and received equivalent volumes of saline. Twenty-four hours after the last administration, the MIRI model was established via LAD artery ligation.

##### Measurement of Serum Levels of Relevant Myocardial Biomarkers in Rats

Following completion of the echocardiography examination, the rats were anesthetized using sodium pentobarbital (administered intraperitoneally). Blood samples were subsequently collected from the abdominal aorta. Blood was centrifuged at 3000 r·min^−1^ for 10 min to separate the serum, which was divided into two portions: one for subsequent testing and the other stored at −80 °C for future use. Serum concentrations of CK-MB and cTn-I were determined using standard assays, from each group using an immunosuppression method, strictly adhering to the kit instructions.

##### Detection of CTRP9, P2Y13, and CCR1 Gene Expression via RT-qPCR

Following drug administration in each group, total RNA purified from tissues was subjected to reverse transcription for cDNA synthesis, according to the manufacturer’s instructions, followed by RT-qPCR analysis. The primers for the CTRP9, P2Y13, and CCR1 genes used in this experiment were synthesized by Shanghai Sangon Biotech Co., Ltd., with their sequences listed in Table 1.

##### Assessment of Cardiac CTRP9, P2Y13, and CCR1 Expression: Western Blot Analysis

Cardiac tissue samples were retrieved from −80 °C storage. A portion of the myocardial tissue samples was collected and weighed; myocardial tissue was minced and homogenized in tissue lysis buffer at a ratio of 10 mg tissue per 100 μL buffer using a handheld tissue homogenizer. The homogenate was further lysed using an ultrasonic cell disruptor and then placed on ice for 20 min. Subsequently, the lysate was centrifuged at 12,000 r·min^−1^ for 15 min, and the supernatant was collected as the total protein extract.

After determining the protein concentration of the sample using the BCA method, we added 5× protein loading buffer and denatured the sample at 100 °C for 5 min in a water bath. We separated 30–50 μg of protein using SDS-PAGE and transferred it to a PVDF membrane. Subsequently, we incubated the protein at room temperature with 5% skim milk for 2 h, then added the primary antibodies (CTRP9, P2Y13, CCR1), and incubated the mixtures overnight at 4 °C.

The following day, the membrane was washed with 1× TBST and incubated with HRP-conjugated secondary antibody for 2 h at room temperature on a shaker. After final washing, the membrane was imaged using a multifunction chemiluminescence imaging system. Densitometric analysis was performed on protein bands using ImageJ, and the relative abundance of target proteins was calculated after normalization to the internal reference.

### 2.10. Statistical Analysis

**Figure 1:**
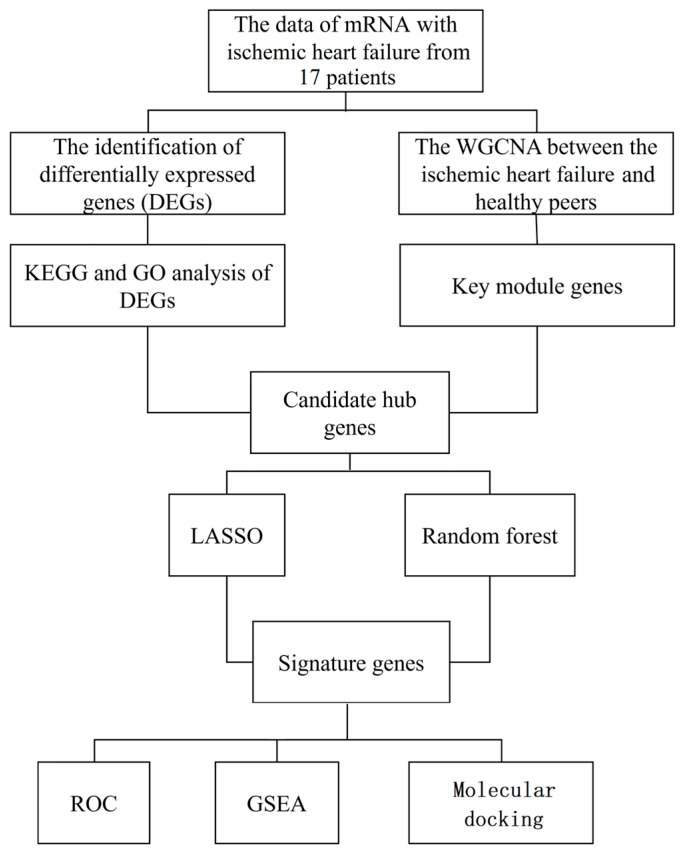
Bioinformatics analysis workflow for mRNA data in patients with IHF.

This study employed R (v4.1.2) and GraphPad Prism (v9.5) for all statistical computations. Data are expressed as the mean ± standard deviation (SD). Intergroup comparisons were conducted using one-way analysis of variance (ANOVA). A *p*-value < 0.05 was considered statistically significant, and all *p*-values represent two-tailed test results. A schematic of the study design is provided in Figure 1.

## 3. Results

### 3.1. HPLC and HRMS Results

**Figure 2:**
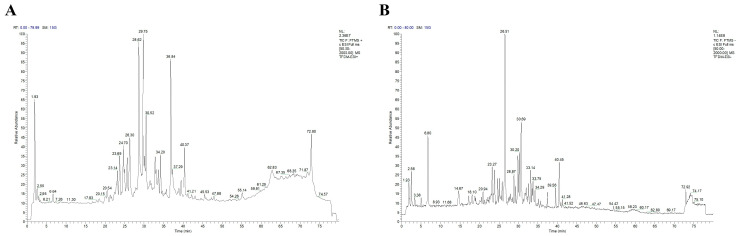
Total ion current (TIC) chromatograms of total flavonoids from *Dracocephalum moldavica* L. (TFDM) acquired via HPLC-HRMS in positive (**A**) and negative (**B**) electrospray ionization (ESI) modes.

The UV spectrum of TFDM showed the characteristic absorption peaks of flavonoid compounds, indicating that TFDM is rich in flavonoid components. The total ion current (TIC) chromatograms of TFDM in positive and negative ion modes (Figure 2) showed multiple peaks, reflecting the diversity of chemical components in TFDM.

### 3.2. Identification of DEGs Between Ischemic Heart Failure Patients and the Control Group

**Figure 3:**
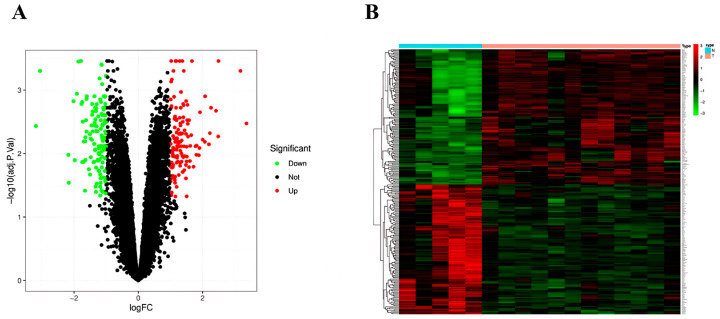
Volcano plot (**A**) and heatmap (**B**) of differentially expressed genes associated with IHF.

Using the “limma” software package to analyze differentially expressed genes between IHF patients and healthy controls, a total of 259 DEGs were identified, including 127 upregulated and 132 downregulated genes (Figure 3A) in the IHF group. Heatmap visualization illustrates the top 50 most significantly up- and downregulated DEGs in the IHG group, respectively (Figure 3B).

### 3.3. Function Enrichment Analysis

**Figure 4:**
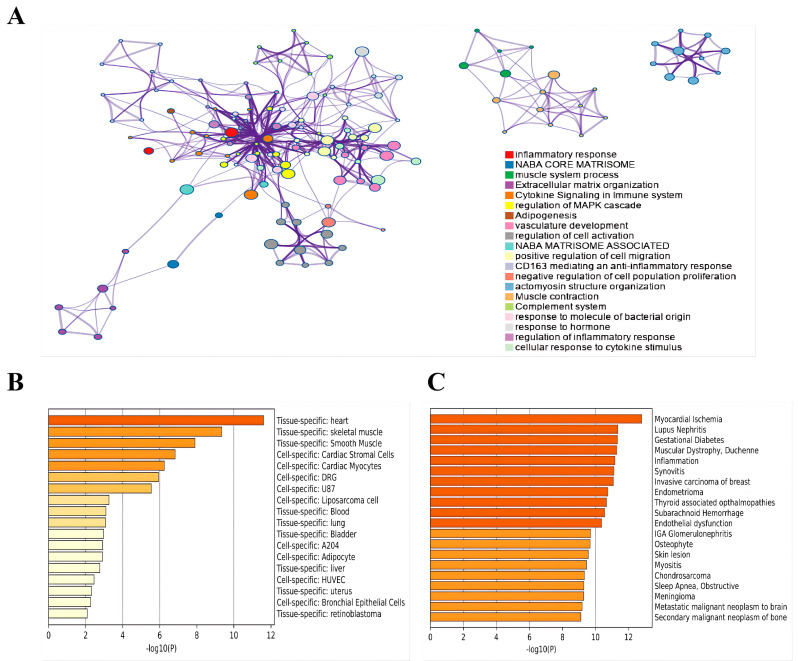
Functional enrichment and association analysis of differentially expressed genes (DEGs). (**A**) Functional enrichment network of DEGs. (**B**) Tissue/cell-specific enrichment analysis of DEGs. (**C**) Disease-associated enrichment analysis of DEGs.

As depicted in Figure 4A, the KEGG analysis revealed that the top enriched pathways included those primarily associated with the inflammatory response, muscle system processes, regulation of the MAPK cascade, vasculature development, orchestrating cell activation, migration and modulation cellular activation, motility, and proliferation. The GO analysis, as shown in Figure 4B, indicates the top three tissue specificities to be the heart, skeletal muscle, and smooth muscle. In terms of cellular specificity, it involves cardiac stromal cells, cardiac myocytes, DRG cells, U87 cells, and liposarcoma cells, among others. The diseases potentially implicated encompass myocardial ischemia, lupus nephritis, gestational diabetes, muscular dystrophy, and inflammation, as illustrated in Figure 4C.

### 3.4. Construction of the Weighted Gene Co-Expression Network

**Figure 5:**
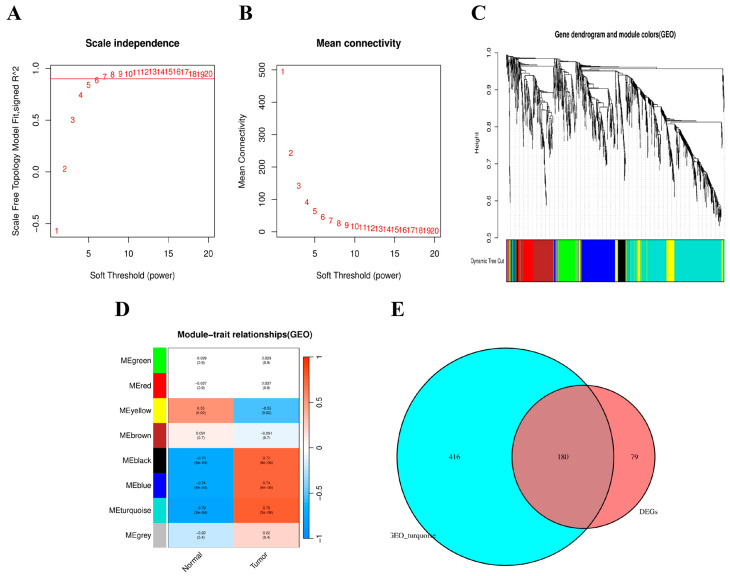
Weighted gene co-expression network analysis (WGCNA) and gene set intersection analysis: (**A**) soft-thresholded scale-free topological fit index analysis; (**B**) soft-thresholded average connectivity analysis; (**C**) gene clustering dendrogram and module partitioning; (**D**) association analysis between modules and phenotypes (normal/disease); (**E**) Venn diagram showing the intersection of GEO dataset genes and differentially expressed genes (DEGs).

Using the WGCNA pipeline, a scale-free co-expression network was constructed for patients with IHF and healthy controls. The soft threshold index was set to 10, yielding a scale-free fit index of 0.85, indicating good average network connectivity (Figure 5A,B). Figure 5C displays the hierarchical clustering dendrogram, with genes clustered into eight modules (Figure 5D). Module–trait association analysis revealed that the MEturquoise module showed a strong negative correlation with IHF (coefficient: r = −0.79, *p* < 0.0002). This module, comprising 595 genes, was identified as a key disease-associated module. Figure 5E presents the overlapping gene set derived from the differential expression analysis and the key co-expression module.

### 3.5. Feature Selection via LASSO and Random Forest

**Figure 6:**
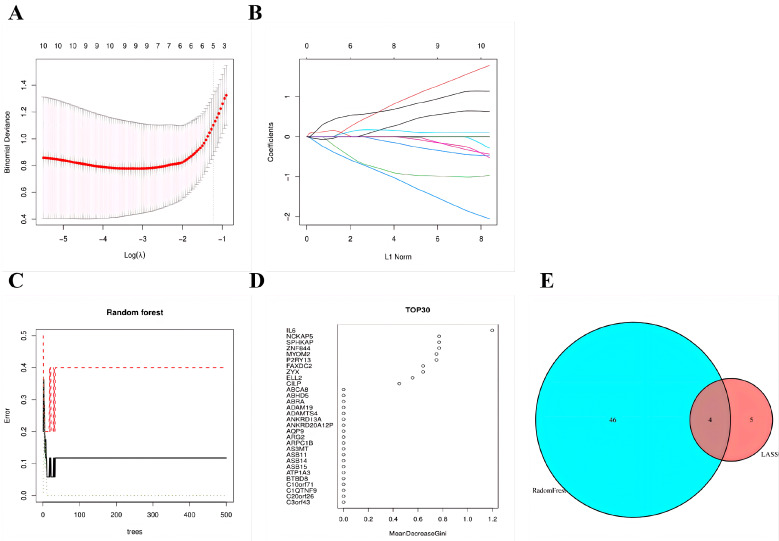
Feature gene screening and intersection analysis. (**A**) LASSO regression bias analysis; (**B**) coefficient change trajectory of LASSO regression; (**C**) error analysis of the Random Forest model; (**D**) average reduction in Gini coefficient for the top 30 genes in Random Forest; (**E**) Venn diagram of genes screened by LASSO and Random Forest.

This study integrated LASSO and the Random Forest algorithm to refine the candidate genes into a robust feature set. LASSO regression identified nine feature genes (Figure 6A,B), and the Random Forest algorithm yielded 50 genes meeting the importance criterion (>0.25) (Figure 6C,D). The intersection of genes identified by both algorithms ultimately yielded four core characteristic genes: C1QTNF9, CCR1, CD163, and P2RY13 (Figure 6E, Table 1).

### 3.6. Diagnostic Utility of Feature Genes in IHF Prediction

**Figure 7:**
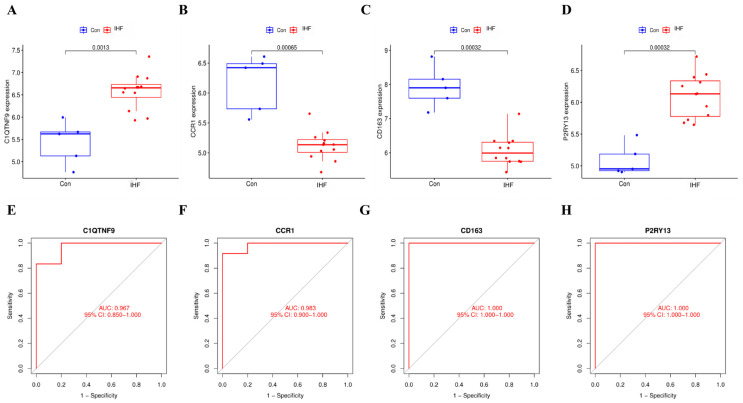
Differential expression of signature genes and diagnostic performance analysis. (**A**–**D**) Box plots of signature gene expression levels in control (Con) versus treated (Treat) groups, demonstrating expression differences and statistical significance for C10TNF9, CCR1, CD163, and P2RY13; (**E**–**H**) ROC curve analysis of signature genes, demonstrating diagnostic performance (AUC values and 95% confidence intervals) for each gene, reflecting their ability to distinguish between the two sample groups.

The selected feature genes exhibited significant differences in expression levels between patients with IHF and healthy individuals (Figure 7A–D), suggesting their potential critical role in disease progression. Further evaluation of the diagnostic efficacy via ROC curves yielded AUC values of 0.967, 0.983, 1.000, and 1.000 for C1QTNF9, CCR1, CD163, and P2RY13, respectively (Figure 7E–H). Collectively, these signature genes exhibit diagnostic value for IHF.

### 3.7. GSEA Results

**Figure 8:**
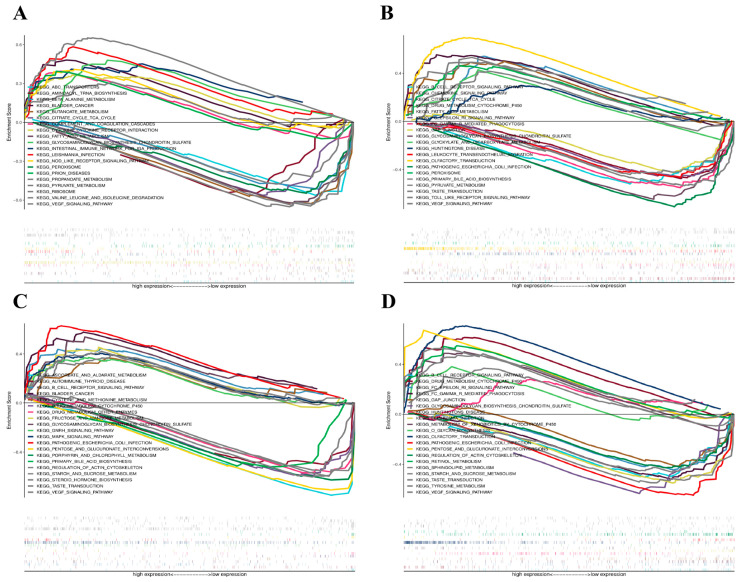
Gene set enrichment analysis (GSEA) results. (**A**–**D**) KEGG pathway enrichment curves across different groups, displaying significantly enriched signaling pathways in the high-expression and low-expression groups. The Enrichment Score reflects the degree of pathway enrichment, with the corresponding heatmap below illustrating the expression distribution of genes within the pathway.

The enrichment of signature genes in specific signaling pathways was assessed using GSEA. Figure 8 displays the top 10 upregulated and downregulated signaling pathways. The results showed that C1QTNF9 is implicated in the following pathways: Vascular Endothelial Growth Factor (VEGF) Signaling Pathway, Valine, Leucine, and Isoleucine Degradation, Ribosome, Pyruvate Metabolism, Propionate Metabolism, Prion Diseases, Peroxisome, NOD-Like Receptor (NLR) Signaling Pathway, Leishmaniasis Infection, Intestinal Immune Network for Immunoglobulin A (IgA) Production, Glycosaminoglycan Biosynthesis: Chondroitin Sulfate, Fatty Acid Metabolism, Cytokine Receptor Signaling, Activation of the complement and coagulation cascades, Citrate Cycle (TCA Cycle), Butanoate Metabolism, Bladder Cancer, Beta-Alanine Pathways governing metabolic activity, tRNA charging, and ABC-mediated efflux. These associations suggest a potential role for C1QTNF9 in a variety of biological processes and disease states.

As shown in Figure 8B, CCR1 expression is associated with the following signaling pathways: VEGF and TLR signaling cascades, taste transduction (previously labeled as TASTE_TRANSDUCTION, now corrected to taste transduction), pyruvate metabolism, Primary Bile Acid Biosynthesis, peroxisomes, pathogenic *E. coli* infection, olfactory transduction, leukocyte transendothelial migration, Huntington’s disease, glycerate and dicarboxylic acid metabolism, glycosaminoglycan biosynthesis: Chondroitin sulfate, Gap junctions, Fcγ receptor-mediated phagocytosis, Fcε receptor signaling pathway, Fatty acid metabolism, Drug metabolism: cytochrome P450, Citric acid cycle (TCA cycle), The chemokine and BCR signaling axes. These pathways regulate diverse cellular functions and disease processes, suggesting CCR1 may play a potential role in mediating these biological mechanisms.

Figure 8C demonstrates that the expression of CD163 is associated with the following signaling pathways: The VEGF signaling axis, Tyrosine Metabolism, Taste Transduction, Starch and Sucrose Metabolism, Sphingolipid Metabolism, Retinol Metabolism, Regulation of Actin Cytoskeleton, Pentose and Glucuronate Interconversions, Pathogenic Escherichia coli Infection, Olfactory Transduction, O-Glycan Biosynthesis, Metabolism of Xenobiotics by Cytochrome P450, Leishmania Infection, Huntington’s Disease, Glycosaminoglycan Biosynthesis: Chondroitin Sulfate, Gap Junction, Fc Gamma R-Mediated Phagocytosis, Fc Epsilon RI Signaling Pathway, Drug Metabolism: Cytochrome P450, B Cell Receptor Signaling Pathway. As a potential regulatory factor in these pathways, changes in the expression of CD163 may have significant impacts on these biological processes.

As shown in Figure 8D, P2RY13 expression is associated with multiple signaling pathways, prominently featuring VEGF signaling, taste transduction, steroid hormone biosynthesis, maltose and sucrose metabolism, actin cytoskeleton regulation, sisprimary bile acid, porphyrin and chlorophyll metabolism, pentose–glucuronic acid interconversion, pathogenic *E. coli* infection, Signaling via the MAPK and GnRH pathways, glycosaminoglycan biosynthesis: chondroitin sulfate, fructose and mannose metabolism, drug metabolism: Other enzymes, drug metabolism: cytochrome P450, cysteine and methionine metabolism, bladder cancer, Activation of BCR signaling and the progression of autoimmune thyroid disease, ascorbic acid and aldol-ketone acid metabolism. As a potential regulator in these pathways, P2RY13 may offer novel strategies and targets for disease prevention and treatment.

### 3.8. Molecular Docking Results of the Effective Compound Components in TFDM with the Proteins

To identify the active components of TFDM, the SwissADME™ analysis platform and a literature review were employed. To ensure data accuracy and completeness, a small number of bioactive molecules that did not meet the screening criteria were included in the candidate active molecules based on the literature reports. Ultimately, 21 candidate active molecules were screened, with the detailed results presented in Table 2.

**Figure 9:**
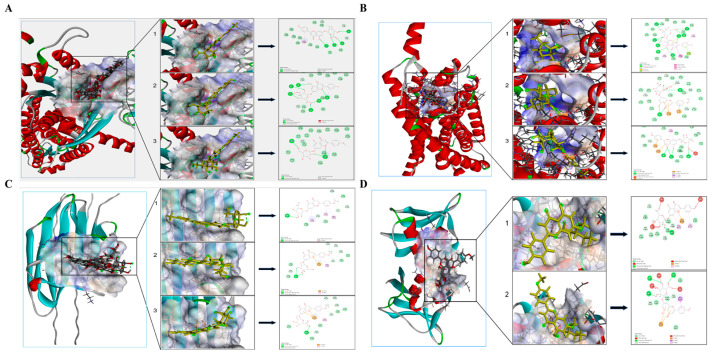
Molecular docking analysis of protein-small-molecule ligands. (**A**–**D**) Docking results for different proteins and candidate compounds. The left panel shows the protein’s 3D structure and docking binding region; the middle panel displays the binding mode between the compound and protein; the right panel presents the compound structure and key interaction sites, reflecting the compound’s binding affinity and mode of action with the protein.

The molecular docking results showed the binding affinities and interaction modes of 21 active components in TFDM with C1QTNF9, P2RY13, CD163, and CCR1, and the results with higher binding forces were visualized, as shown in Figure 9A–D.

### 3.9. Experimental Verification

#### 3.9.1. Effects of TFDM on the Levels of Myocardial Injury Biomarkers in Rats

**Figure 10:**
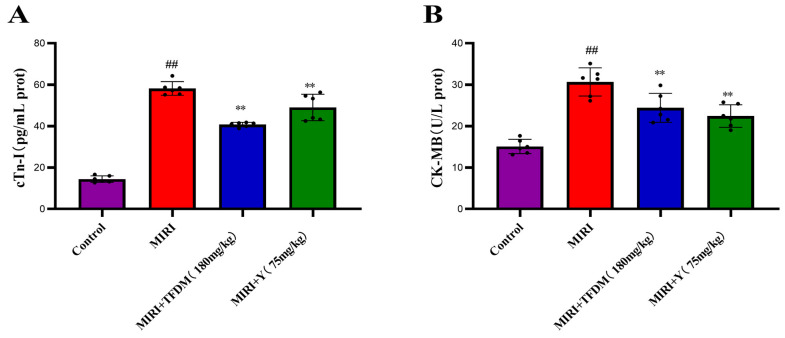
Changes in myocardial injury markers across different groups. (**A**) Serum cardiac troponin I (cTnI) levels; (**B**) Serum creatine kinase isoenzyme (CK-MB) levels. Data are presented as mean ± SD. ## *p* < 0.01 vs. the control group; ** *p* < 0.01 vs. the MIR model group.

The levels of cTn-I and CK-MB are routinely employed to diagnose myocardial injury. The serum myocardial biomarker assay results (Figure 10) showed that, compared with the control group, the serum levels of cTn-I and CK-MB were significantly increased in the model group (*p* < 0.01). In comparison with the model group, both the TFDM group and the positive drug group significantly reduced the serum levels of cTn-I and CK-MB, and the differences were statistically significant (*p* < 0.01). These results suggest that TFDM can attenuate myocardial injury in rats with MIRI.

#### 3.9.2. Effects of TFDM on the mRNA Expression of the CTRP9, P2Y13, and CCR1 Genes

**Figure 11:**
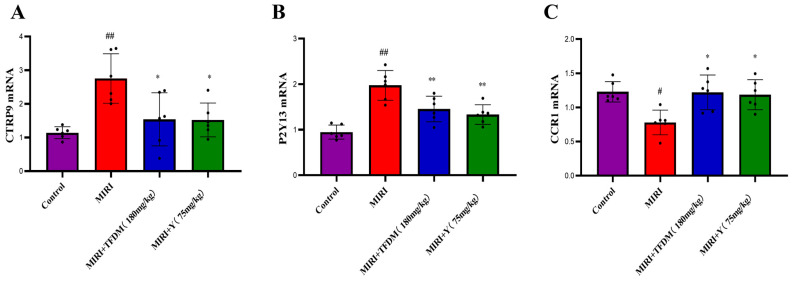
mRNA expression levels of target genes in different treatment groups. (**A**) C1QTNF9 mRNA levels: Expression was significantly elevated in the MIRI group, while MIRI + TFDM and MIRI + Y treatments downregulated its expression. (**B**) P2RY13 mRNA levels: The trend was consistent with C1QTNF9; the MIRI group showed upregulation, while the intervention groups showed reduced expression levels. (**C**) CCR1 mRNA levels: The MIRI group showed reduced expression, while the MIRI + TFDM and MIRI + Y treatments restored expression. Note: # and ## indicate significant differences from the control group ( # *p* < 0.05, ## *p* < 0.01); * and ** indicate significant differences from the MIRI group (* *p* < 0.05, ** *p* < 0.01), reflecting statistical significance in expression levels between groups.

In this study, RT-qPCR analysis revealed that under the treatment of TFDM and the positive drug group, the mRNA expressions of CTRP9 and P2Y13 were significantly upregulated in a dose-dependent manner, while the mRNA expression of CCR1 was obviously downregulated in a dose-dependent manner. The results (Figure 11) indicated that TFDM may be involved in regulating the related myocardial cell signaling pathways by specifically activating the expressions of CTRP9 and P2Y13 and inhibiting the expression of CCR1.

#### 3.9.3. Effects of TFDM on the Protein Expression of CTRP9, P2Y13, and CCR1 in Cardiac Tissue

**Figure 12:**
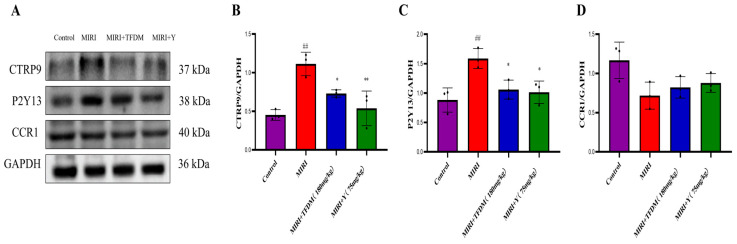
Expression levels of the target proteins in different treatment groups. (**A**) Western blot results: protein bands for CTRP9, P2Y13, CCR1, and the housekeeping gene GAPDH (labeled with corresponding molecular weights). (**B**) Relative expression levels of CTRP9 protein, which were significantly upregulated in the MIRI group; MIRI + TFDM and MIRI + Y treatments reduced its expression. (**C**) Relative expression levels of P2Y13 protein: The trend was consistent with CTRP9: elevated in MIRI group and downregulated in the intervention groups. (**D**) Relative expression of CCR1 protein, which was decreased in the MIRI group and restored in MIRI + TFDM and MIRI + Y treatments (Note: ## indicates a statistically significant difference from the control group; ** indicates an extremely statistically significant difference from the control group; * indicates a statistically significant difference from the MIRI group, reflecting the statistical significance of protein levels between groups).

The results (Figure 12) suggest that TFDM may exert cardioprotective effects by regulating the expression of CTRP9, P2Y13, and CCR1, thereby modulating related signaling pathways or biological processes in myocardial tissue.

## 4. Discussion

The clinical syndrome of IHF entails structural or functional abnormalities of the heart. Its pathophysiological mechanism is manifested as impaired ventricular contractility or diastolic function, leading to reduced cardiac output or elevated intracardiac pressure. Common symptoms include dyspnea, peripheral edema, and palpitations accompanied by insomnia. Early diagnosis and treatment are crucial for improving the prognosis and rehabilitation outcomes of patients with IHF.

In this study, we first performed qualitative profiling of TFDM using HPLC-HRMS technology to clarify its chemical composition. The results showed that TFDM contains a variety of chemical components, including flavonoids such as acacetin, apigenin, luteolin, quercetin, and kaempferol, as well as other compounds. These results are consistent with the known chemical composition characteristics of *Dracocephalum moldavica* L. and also provide a material basis for subsequent studies on the mechanism of TFDM in the treatment of IHF.

Subsequently, WGCNA was employed to evaluate the differentially expressed genes (DEGs) between patients with IHF and healthy controls and to identify key gene modules. Least Absolute Shrinkage and Selection Operator (LASSO) regression and the Random Forest algorithm were applied to screen IHF-related signature genes, including C1QTNF9, CCR1, CD163, and P2Y13. ROC curve analysis showed that these four genes have high diagnostic value for IHF, with AUC values all above 0.96, indicating that they have the potential to be used as candidate diagnostic biomarkers for IHF.

CTRP9 is predominantly sourced from myocardial capillary endothelial cells. The expression of CTRP9 mRNA is found to be upregulated in hypertrophic human hearts and in mouse hearts subsequent to transverse aortic constriction (TAC). Additionally, increased levels of CTRP9 protein are observed in the serum of patients suffering from severe aortic stenosis and in murine hearts post-TAC. Notably, mice with either heterozygous or, particularly, homozygous ablation of the C1qtnf9 (CTRP9) gene displayed a protective effect against the onset of cardiac hypertrophy, left ventricular dilation, and subsequent heart dysfunction during TAC [17]. These findings suggest that CTRP9 may emerge as a potential therapeutic target for future clinical interventions.

CCR1 is expressed on vascular endothelial cells, with its primary ligands being CCL3 and CCL5; these chemokines are predominantly released by activated platelets. CCR1 and its ligands primarily mediate the infiltration of inflammatory cells [18,19]. In a myocardial infarction model of mice with CCR1 knockout, there was a reduction in neutrophil infiltration and an increase in monocyte infiltration. Additionally, there was an increase in collagen synthesis and myofibroblast production, which significantly alleviated myocardial injury and necrosis, promoted scar healing, and improved ventricular remodeling [20].

CD163 is specifically expressed on the surface of monocyte–macrophages and is recognized as a hemoglobin scavenger receptor [21,22,23,24], which is believed to play roles in reducing lipid peroxidation, exerting anti-inflammatory effects, and combating atherosclerosis. CD163 mediates the clearance of free hemoglobin (FHb) by monocytes/macrophages, thereby playing anti-lipid peroxidative and anti-inflammatory roles. CD163 can specifically recognize and clear the hemoglobin–haptoglobin (Hb-Hp) complex, facilitating its internalization into monocytes/macrophages, where the heme component of hemoglobin is degraded to biliverdin, iron ions (Fe^2+^), and carbon monoxide (CO) under the action of heme oxygenase-1 (HO-1) [25]. Fe^2+^ is further incorporated into ferritin, which prevents the release of Fe^2+^ that could cause tissue oxidative damage. Schaer et al. demonstrated that in the absence of haptoglobin, free hemoglobin (FHb) directly interacts with CD163 and undergoes degradation in macrophages via receptor-mediated endocytosis. This macrophage-mediated scavenging pathway is critical for preventing hemoglobin-induced oxidative stress and tissue injury. Consistently, tissue-resident macrophages have been shown to protect the heart under stress conditions by maintaining cardiomyocyte homeostasis [21], while CD163 itself has been identified as a valuable diagnostic marker for inflammatory disorders [22]. CD163 can also directly modulate the secretion of cellular inflammatory factors (such as IL-10), antioxidant factors (like HO-1), and granulocyte-macrophage colony-stimulating factor via intracellular signal transduction, playing a direct role in the anti-inflammatory process [26].

Research has found that CD163 is closely associated with cardiac function. Logistic regression analysis has shown that, after adjusting for age, hypertension, diabetes, history of stroke, systolic pressure, diastolic pressure, hemoglobin, creatinine, and other risk factors, CD163 still significantly correlates with chronic heart failure (CHF). CD163 is strongly associated with the severity indicators of heart failure such as CRP, IL-1β, IL-6, HO-1, and MDA, indicating that CD163, along with other inflammatory factors [27], is involved in the pathophysiological processes of heart failure and is a characteristic marker of its etiology.

P2Y13 is a G protein-coupled receptor (GPCR) from the P2Y subfamily. Similarly to P2Y12 and P2Y14, it can be activated by adenosine diphosphate (ADP). First, this receptor exhibits multifaceted signaling capabilities. Beyond the classical G protein pathway, it can couple with multiple G proteins including Gs and Gq, thereby activating key intracellular signaling axes such as MAPKs, PI3K/Akt/GSK3. Concurrently, studies in P2Y13 knockout mice indicate its specific role in cholesterol and glucose metabolism, as well as bone homeostasis maintenance. Furthermore, this receptor participates in regulating multiple central nervous system functions, such as pain transmission and neuroprotection. However, the role of P2Y13 in IHF remains unclear and warrants further investigation [28].

The molecular docking results showed that multiple active components in TFDM (such as quercetin, luteolin, kaempferol) have strong binding affinity with the proteins encoded by the four characteristic genes. However, molecular docking only suggests potential intermolecular interactions, rather than confirming a direct causal relationship between binding and biological function. Nevertheless, these in silico results support the rationale that TFDM may exert its therapeutic effect on IHF by targeting these proteins. In vivo experiments further confirmed that TFDM can reduce the levels of the serum myocardial injury markers cTn-I and CK-MB in MIRI model rats, upregulate the mRNA and protein expressions of CTRP9 and P2Y13, and downregulate the expression of CCR1. These results indicate that TFDM can alleviate myocardial ischemia–reperfusion injury through multiple pathways, which is consistent with the results of the molecular docking.

Research conducted by our team over the years has demonstrated that the total flavonoids of *Dracocephalum moldavica* (TFDM) possess pharmacological effects against myocardial and cerebral ischemia–reperfusion injury [29,30,31,32,33,34,35,36]. However, the unclear mechanisms of action and the undefined active components present significant challenges that severely hinder their widespread application. To address the urgent clinical treatment needs and overcome the bottleneck in this traditional Chinese medicine therapy, this study adopted a research model that combined bioinformatics, qualitative analysis, and machine learning. Systematic analysis and detailed research were conducted at different levels, including genes, proteins, and chemical components. Molecular docking showed that the active components in TFDM have a strong binding affinity to the target indicators, which can lay the foundation for further drug development.

This study has several limitations that warrant in-depth clarification. First, as all data were derived from public repositories with a relatively small sample size, selection bias and limited generalizability may exist. Second, the machine learning models lacked an independent external validation cohort, which may increase the risk of overfitting despite the high AUC values observed. Third, the HPLC-HRMS analysis was qualitative rather than quantitative, which limits the interpretation of the dose–effect relationship and precise pharmacological contribution of each component. Fourth, the bioinformatic analysis was based on chronic IHF patients, whereas the experimental validation was performed in an acute MIRI rat model; the translational difference between chronic and acute conditions requires further justification. Finally, whether the immune cell infiltration associated with core genes correlates with the progression of IHF also requires further research.

In conclusion, this study clarified the chemical composition of TFDM through qualitative analysis and then identified four signature genes—C1QTNF9, CCR1, CD163, and P2Y13—that show potential preliminary value in the early diagnosis of IHF. Furthermore, we explored the binding affinity between the protein targets of these signature genes and active components in TFDM, supported by in vivo validation in rats. This study provides new insights for the diagnosis and treatment of IHF and lays a preliminary foundation for the development of TFDM as a potential clinical agent for IHF. However, all findings remain preliminary and require further confirmation by independent clinical cohorts and rigorous mechanistic studies before clinical translation.

## Figures and Tables

**Table 1:** Primer sequences.

Gene	Primer Sequence F (5′-3′)	Primer Sequence R (5′-3′)
CTRP9	GCAGGTGACAGGAGGAGAGAGG	TGAACAGAAGGAAGCCCGTGAATG
P2Y13	GCGTGGCAAGGACTGTGAAGTG	TCAGGGCATGTGCAGAAAGTTAGC
CCR1	ACCCAATGAGAAGAAGGCCAAAGC	TGCTTGCTCTGCTCACACTGATTG
GAPDH	CTCTCTGCTCCTCCCTGTTC	TCACACCGACCTTCACCATC

**Table 2:** Active compounds screened from TFDM.

NO.	Compound Name	PubChem CID
HT01	8-hydroxy-salvigenin	3083783
HT02	scrophulein	188323
HT03	acacetin	5280442
HT04	apigenin	5280443
HT05	luteolin	5280445
HT06	chrysoeriol	5280666
HT07	diosmetin	5281612
HT08	gardenin A	261859
HT09	gardenin B	96539
HT10	isorhamnetin	5281654
HT11	kaempferol	5280863
HT12	quercetin	5280343
HT13	salvigenin	161271
HT14	syringaresinol	100067
HT15	3-hydroxyflavone	11349
HT16	moldavoside, acacetin 7-O-glucoside	5321954
HT17	apigenin-7-O-β-D-galactoside	44257799
HT18	acacetin7-O-β-D-glucopyranoside	44257884
HT19	acacetin-7-O (-6″-acetyl) -glucopyranoside	52929806
HT20	acacetin-7-O-β-D-glucuronide	44257886
HT21	apigenin-7-O-β-D-glucoside	5280704

## Data Availability

The original contributions presented in this study are included in the article. Further inquiries can be directed to the corresponding authors.
